# Potential Bud Bank Responses to Apical Meristem Damage and Environmental Variables: Matching or Complementing Axillary Meristems?

**DOI:** 10.1371/journal.pone.0088093

**Published:** 2014-02-06

**Authors:** Jitka Klimešová, Lenka Malíková, Jonathan Rosenthal, Petr Šmilauer

**Affiliations:** 1 Department of Functional Ecology, Institute of Botany, Academy of Sciences of the Czech Republic, Třeboň, Czech Republic; 2 Department of Botany, Faculty of Science, University of South Bohemia, České Budějovice, Czech Republic; 3 Department of Biological Sciences, State University of New York Ulster, Stone Ridge, New York, United States of America; 4 Department of Ecosystem Biology, Faculty of Science, University of South Bohemia, České Budějovice, Czech Republic; USDA-ARS, United States of America

## Abstract

Soil nutrients, dormant axillary meristem availability, and competition can influence plant tolerance to damage. However, the role of potential bud banks (adventitious meristems initiated only after injury) is not known. Examining Central European field populations of 22 species of short-lived monocarpic herbs exposed to various sources of damage, we hypothesized that: (1) with increasing injury severity, the number of axillary branches would decrease, due to axillary meristem limitation, whereas the number of adventitious shoots (typically induced by severe injury) would increase; (2) favorable environmental conditions would allow intact plants to branch more, resulting in stronger axillary meristem limitation than in unfavorable conditions; and (3) consequently, adventitious sprouting would be better enabled in favorable than unfavorable conditions. We found strong support for the first hypothesis, only limited support for the second, and none for the third. Our results imply that whereas soil nutrients and competition marginally influence plant tolerance to damage, potential bud banks enable plants to overcome meristem limitation from severe damage, and therefore better tolerate it. All the significant effects were found in intraspecific comparisons, whereas interspecific differences were not found. Monocarpic plants with potential bud banks therefore represent a distinct strategy occupying a narrow environmental niche. The disturbance regime typical for this niche remains to be examined, as do the costs associated with the banks of adventitious and axillary reserve meristems.

## Introduction

Short-lived monocarpic plants that lose apical meristems are able, to varying degrees, to compensate or even overcompensate (i.e. increase their fitness relative to intact individuals) for biomass loss [1], [2], [3]. Compensation has been shown to decrease with increasing plant injury, as limitation by axillary meristem availability becomes more important [4]. However, compensatory growth can occur not just by axillary branching, but also by the activation of adventitious meristems, which largely serve as a potential bud bank (*sensu* [5]).

Seeking to discern the relationship between environmental variables and compensatory responses to herbivory, different models have yielded radically different predictions of the effects of nutrient availability and resource competition on compensatory responses to apical meristem damage (AMD). Thus, the compensatory continuum hypothesis (CCH) generally predicts that compensation should increase with resource availability [6], whereas the growth rate model (GRM) predicts that it would decrease [7], with neither particularly successful at predicting outcomes [8], [9]. The limiting resources model (LRM), when configured to consider meristems, along with other requirements (e.g., nutrients, water) as resources, is much more successful [10], although this depends upon the ability of researchers to identify the actual limiting resource, which is context-dependent (see critique by Banta *et al.* [11]). Another line of theoretical research has attempted to identify favourable strategies for activating meristems in response to disturbance regimes of different frequencies or intensities (e.g., [12], [13]).

One limitation of AMD experimental studies is that they have all been done at the species level, ignoring potential ecosystem-level responses which could comprise species displacement along productivity and disturbance gradients. A model for community-level responses of disturbance-tolerant plants has been constructed by Bellingham and Sparrow [14], but it focuses primarily, although not exclusively, on fire responses of woody plants.

As noted above, much of the theoretical investigation of compensatory growth responses to AMD has been predicated on the assumption that they have evolved due to selection pressure from herbivory. Moreover, this assumption has underlain field and experimental investigations of such responses in short-lived monocarps, with the latter typically featuring AMD inflicted in ways that mimic damage from herbivores. In focusing largely on herbivory as the source of AMD, theoretical and empirical research has paid scant attention to other causes. These causes can include fire, trampling, desiccation, disease, erosion, and numerous anthropogenic activities [15], [16]. Indeed, we suggest that one evolutionary advantage that compensatory growth might have over herbivory avoidance (via chemical or physical defenses or phenological escape) as an herbivory coping strategy is that it likely helps plants cope with multiple possible sources of AMD.

With few exceptions, AMD investigations of short-lived polycarpic herbs have focused on axillary meristems (whereas other structures have been considered in studies of woody plants). Thus, they have largely overlooked the fact that some plants can respond to damage by generating adventitious meristems, which largely serve as a potential bud bank (*sensu* [5]). Indeed, adventitious sprouting is usually triggered by severe injury to the plant body [17]. Field studies and pot experiments on the impacts of environmental characteristics such as light, soil nitrogen, moisture, and herb layer cover on adventitious sprouting have produced disparate results, with the former [16] revealing an absence of effects, and the latter [18], [19] finding significant influences.

Vesk and Westoby [20] bemoaned the lack of quantitative data on sprouting generally. Some of the present authors, in a previous paper [16], attempted to at least partially fill that gap by quantifying adventitious sprouting for 22 short-lived monocarpic species occurring in numerous field sites and related these responses to damage severity as well as environmental characteristics and plant size. In the present study, we build on these findings to generate a more integrated understanding of compensatory responses in these species, and in particular to elucidate the relationship between axillary sprouting and adventitious sprouting.

At the study’s core, we examine the axillary meristem responses of these plants in relationship to the same damage, environmental and plant size variables and statistically compare them with their adventitious sprouting responses. Moreover, we employ multiple approaches that can enable us to garner fresh insights into several aspects of compensatory responses. Firstly, based on the recognition that adventitious sprouting is often triggered by severe plant damage, we wish to determine whether the damage threshold for initiating this sprouting is higher than that for axillary sprouting. Second, since, as Vesk and Westoby [21] suggested generally, different preformed buds incur different costs, we could expect that the different types of sprouting would differ in their relationships to environmental variables and plant sizes. Third, by comparing the responses of damaged and undamaged plants to the environmental variables, we can obtain crucial information in determining whether a particular resource is limiting, *sensu* Wise and Abrahamson [22], and how the AMD affects the ability to exploit that resource. Fourth, analyzing the data both within individual species (across populations) and across species, allows us to assess the generality of both physiological responses at both these levels, with the within-species comparisons enabling us to determine the context-dependency of the responses. Finally, to account for possible evolutionary conservatism, we perform the analyses both with and without phylogenetic correction, which will also allow us to infer whether the compensatory responses are phylogenetically labile, as has been reported by Vesk and Westoby [20], [21].

By examining numerous species across many sites (yielding a total of 389 populations) and considering both axillary and adventitious meristem responses in light of plant and environmental characteristics, we hope to achieve a broader understanding of AMD responses. This is especially true because we study these responses in field settings in which the plants are likely to experience various forms of AMD reflecting the varied challenges that many plants face, rather than simply contending with herbivory.

Our specific hypotheses are:

1. With increasing plant injury severity, branching will decrease due to axillary meristem limitation, whereas sprouting will increase due to greater activation of adventitious meristems;
2. Growth of plants in natural communities is limited by nutrients and therefore we expect that axillary branching in intact plants will be greater in favorable (low competition, high light, moisture, and nutrient availability) than adverse conditions. Consequently, in favorable conditions, less axillary meristems will be available for post-injury branching, with injured plants showing less of a branching response than in adverse conditions.
3. Injury will be required to initiate adventitious sprouting, but among injured plants, those in more favorable conditions will be able to support more vigorous sprouting.

## Materials and Methods

### Sampling

In the field, we sampled 22 species of annual or biennial monocarpic herbs that have been reported as being capable of adventitious sprouting from the hypocotyl and/or roots [23], [24] (Appendix S1). None of the species is endangered or protected by law. They were sampled from a total of 389 populations in publicly accessible habitats and various environmental conditions in Central Europe, Czech Republic (mainly in vicinity of towns Praha, České Budějovice, Ústí nad Labem, Třeboň, Veselí nad Moravou), between 2005 and 2007 (see list of studied species, and numbers of sampled populations and individuals in Appendix S1). Sampled habitats included paths, railway stations and embankments, ruderal meadows and pastures, road margins, brook/river banks, construction sites, and arable fields and their margins; no specific permissions were required for these locations. Thus, in addition to likely suffering some herbivory from insects and/or mammals, the plants would have experienced various forms of anthropogenic mechanical damage, and, in the case of brook/river banks, flooding. Plant growth variables assessed were plant height, number of axillary branches, cumulative length of adventitious shoots, number of adventitious buds, and number of adventitious shoots.

Injury severity was assessed according to visible signs of damage on the main shoot in from 20 to 60 (depending roughly on population size) randomly selected plant individuals from each site at which the species occurred. Injury was classified as either low (part of the aboveground biomass had been removed but the part of the stem(s) with axillary buds was preserved), medium (most of the aboveground biomass had been removed, with only a few or no axillary buds preserved but cotyledons and hypocotyl remaining intact), or high (entire aboveground biomass including cotyledons had been removed, with only part of the hypocotyl or roots remaining intact).

The plant community found at each site was described using phytosociological relevés [25], with all species in a relevé (usually 4×4 m) identified and their cover estimated (Braun–Blanquet scale r – 0.05–0.5%, + − 0.5–2.5%, 1–2.5–7.5%, 2a – 7.5–15%, 2m - 15–22.5%, 2b - 22.5–37.5%, 3–37.5–62.5%, 4–62.5–87.5%, 5–87.5–100%). Thus, for each community, total vegetation cover and cover of individual species were also evaluated.

Environmental characteristics of the studied communities, and thus their component populations, were assessed using Ellenberg indicator values (EIVs) for light, moisture and nitrogen. The EIVs, which are based on empirical assessment of preferences of species along environmental gradients, were taken from Ellenberg [26]. The scales for EIVs are ordinal, and generally range from 1 (low end of the gradient) to 9 (high end), but in the case of moisture range from 1 to 12. For our study, the values of the environmental characteristics for each locality (and therefore each of the individual populations sampled there) were calculated as the averages of EIVs for the species occurring in the phytosociological relevé, weighted by the species’ estimated abundance.

### Data Analysis

For the studied environmental characteristics, because we expected that plant responses might not be monotonic and might substantially deviate from a symmetric second-order polynomial form, we replaced the (semi-)quantitative estimates of each environmental characteristic with a classification of species into three groups of approximately the same size, i.e. the third of the species having the highest values for that variable, the third with the lowest values, and the third with intermediate values. This approach provides sufficient resolution for the questions addressed and avoids the use of less commonly adopted approaches such as generalized additive models. Additionally, the resulting descriptors can be very easily included within interaction terms that can be interpreted in a straightforward manner.

We tested our hypotheses using linear mixed-effect models or generalized mixed-effect models (assuming quasi-Poisson distributions), depending on the kind of response variable, with population and species identity treated as random effects for intra-specific analyses, and species identity as the only random effect for inter-specific analyses (for each species, separate averages calculated for injured and intact plants). When comparing the effects of explanatory variables upon injured and intact plants, we looked at their independent (marginal) effects within each group, and also fitted a model comparing the response between these two groups using an interaction term including injury status. Hypothesis tests used likelihood-ratio statistics, comparing the differences in model deviances with a χ^2^ distribution. All models were fitted using the *lme4* package in R, version 2.8 [27].

To take into account possible phylogenetic conservatism in the traits, we applied phylogenetic correction using the method of Desdevises *et al.* [28] where appropriate. This did not include the intra-specific models for plant height and number of axillary branches, from which among-species differences were removed, so that there was no phylogenetic signal left.

## Results

### Intraspecific Level

Competition (herb layer cover), light availability, and soil conditions affected assessed growth variables of injured and intact plants differently (see difference values listed in Tables 1–4), although relationships were seldom significant when evaluating effects of studied factors on injured and intact plants separately. Intact plants showed height increasing with herb layer cover (Table 1), fewer and shorter adventitious shoots and fewer adventitious buds with increasing light availability, and more adventitious shoots and buds with increasing moisture and soil nutrients (Tables 3, 4). The effects of moisture and nutrients disappeared after phylogenetic correction, indicating that the trend was caused by similar responses of closely related species due to evolutionarily conserved traits.

**Table 1:** Effects of environmental characteristics upon plant height.

	Herb layer cover		Light		Soil moisture		Nutrient availability	
	effect	test	effect	test	effect	test	effect	test
**Intact**	▴	28.7 (<1e-6)	–	4.42 (NS)	–	3.32 (NS)	–	3.08 (NS)
**Injured**	▴	7.96 (0.0187)	–	3.95 (NS)	–	1.02 (NS)	–	0.00 (NS)
**Difference**	–	1.35 (NS)	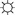	55.6 (<1e-6)	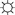	17.4 (0.0002)	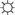	5.40 (0.0672)

Effects of individual predictors (major columns) were assessed separately for intact plants, injured plants, and for the difference between injured and intact plants (as an interaction). For the intact and injured plants, the response of plant height to increasing values of a predictor is presented graphically using up and down arrows, with the 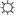 symbol indicating a significant interaction term further characterized in the text. The test statistic is a likelihood ratio to be compared with a χ^2^ distribution with two degrees of freedom. Type I error estimate (significance) is shown in parentheses for values below 0.1, with others shown as NS.

**Table 2:** Effects of environmental characteristics upon the number of axillary branches.

	Herb layer cover		Light		Soil moisture		Nutrient availability	
	effect	test	effect	test	effect	test	effect	test
**Intact**	–	1.16 (NS)	–	0.77 (NS)	–	0.05 (NS)	–	2.28 (NS)
**Injured**	▴	6.32 (0.0425)	–	0.22 (NS)	–	0.30 (NS)	–	2.98 (NS)
**Difference**	–	4.04 (NS)	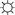	7.58 (0.0226)	–	4.14 (NS)	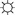	12.5 (0.0019)

Effects of individual predictors (major columns) were assessed separately for intact plants, injured plants, and for the difference between injured and intact plants (as an interaction). For the intact and injured plants, the response of plant height to increasing values of a predictor is presented graphically using up and down arrows, with the 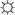 symbol indicating a significant interaction term further characterized in the text. The test statistic is a likelihood ratio to be compared with a χ^2^ distribution with two degrees of freedom. Type I error estimate (significance) is shown in parentheses for values below 0.1, with others shown as NS.

**Table 3:** Effects of environmental characteristics upon the number of adventitious buds and shoots.

		Herb layer cover		Light		Soil moisture		Nutrient availability	
		effect	test	effect	test	effect	test	effect	test
**Intact**	corrected	–	2.24 (NS)	▾	9.50 (0.0087)	–	2.40 (NS)	–	0.78 (NS)
	non-corrected	–	0.79 (NS)	▾	26.8 (1.5e-6)	▴	15.8 (0.0004)	▴	17.8 (0.0001)
**Injured**	corrected	▾	10.8 (0.0045)	–	3.89 (NS)	–	0.62 (NS)	–	0.63 (NS)
	non-corrected	–	1.61 (NS)	▾	10.1 (0.0066)	–	0.93 (NS)	–	1.02 (NS)
**Difference**	corrected	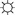	79.0 (<1e-6)	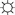	77.1 (<1e-6)	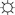	64.8 (<1e-6)	–	0.13 (NS)
	non-corrected	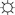	79.2 (<1e-6)	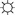	77.0 (<1e-6)	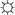	64.9 (<1e-6)	–	0.09 (NS)

Effects of individual predictors (major columns) were assessed separately for intact plants, injured plants, and for the difference between injured and intact plants (as an interaction). For the intact and injured plants, the response of plant height to increasing values of a predictor is presented graphically using up and down arrows, with the 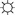 symbol indicating a significant interaction term further characterized in the text. The test statistic is a likelihood ratio to be compared with a χ^2^ distribution with two degrees of freedom. Type I error estimate (significance) is shown in parentheses for values below 0.1 (with others shown as NS), and the results shown in *corrected* rows represent models incorporating phylogenetic correction.

**Table 4:** Effects of environmental characteristics upon cumulative length of adventitious shoots.

		Herb layer cover		Light		Soil moisture		Nutrient availability	
		effect	test	effect	test	effect	test	effect	test
**Intact**	corrected	–	1.28 (NS)	▾	5.79 (0.0551)	–	0.93 (NS)	–	4.37 (NS)
	non-corrected	–	1.30 (NS)	▾	17.5 (0.0002)	–	1.36 (NS)	–	2.59 (NS)
**Injured**	corrected	–	2.49 (NS)	–	0.56 (NS)	–	1.21 (NS)	–	1.73 (NS)
	non-corrected	–	1.34 (NS)	▾	17.0 (0.0002)	–	0.18 (NS)	–	2.72 (NS)
**Difference**	corrected	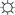	33.7 (<1e-6)	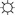	211.1 (<1e-6)	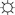	5.28 (0.071)	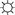	15.6 (0.0004)
	non-corrected	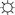	31.6 (<1e-6)	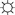	208.2 (<1e-6)	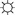	6.53 (0.0383)	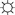	12.3 (0.0021)

Effects of individual predictors (major columns) were assessed separately for intact plants, injured plants, and for the difference between injured and intact plants (as an interaction). For the intact and injured plants, the response of plant height to increasing values of a predictor is presented graphically using up and down arrows, with the 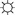 symbol indicating a significant interaction term further characterized in the text. The test statistic is a likelihood ratio to be compared with a χ^2^ distribution with two degrees of freedom. Type I error estimate (significance) is shown in parentheses for values below 0.1 (with others shown as NS), and the results shown in *corrected* rows represent models incorporating phylogenetic correction.

Injured plants also increased in height with increasing herb layer cover (Table 1), with increasing cover values also associated with more axillary branches (Table 2) and fewer adventitious shoots (Table 3). Injured plants were affected by light availability in the same direction as intact plants, producing fewer and shorter adventitious shoots with higher light availability (Table 3, 4). This relationship disappeared after phylogenetic correction due to similarities within entire groups of related species.

Adventitious sprouting differed from axillary branching in its responses to light availability and degree of injury. With greater light availability, plants produced fewer and shorter adventitious shoots. Increasing injury severity was associated with fewer axillary branches but more numerous adventitious shoots (Table 5).

**Table 5:** Effects of disturbance severity upon numbers of axillary branches and adventitious buds and shoots.

		Disturbance severity	
		effect	test
**Axillary branches**	non-corrected	▾	72.7 (<1e-6)
**Buds and shoots**	corrected	▴	6.36 (0.0117)
	non-corrected	▴	6.55 (0.0105)

The responses of the numbers of axillary branches and buds and shoots to increasing severity of disturbance are presented graphically using up and down arrows. The test statistic is a likelihood ratio to be compared with a χ^2^ distribution with one degree of freedom. Type I error estimate (significance) is shown in parentheses and the result shown in *corrected* row represents a model incorporating phylogenetic correction. The number of axillary branches character does not require correction, as it lacks phylogenetic signal (see Methods).

### Interspecific Level

Species inhabiting different parts of environmental gradients differed only marginally in plant height (higher in nutrient-poor than in moderately rich soils, χ^2^_2_ = 6.55, p = 0.0377 without phylogenetic correction and χ^2^_2_ = 10.12, p = 0.0063 with correction) and branching (more branched species were in conditions with high light availability, χ^2^_2_ = 8.08, p = 0.0176 without phylogenetic correction and χ^2^_2_ = 4.13, p = 0.127 with correction). Other parameters (number of adventitious shoots and their length) were not affected by any environmental parameter examined (nutrient availability, light, moisture). We also did not find any effect of injury severity on responses of plant species to environmental gradients.

## Discussion

### General Pattern

Our study is the first to demonstrate that even when axillary branching is limited by availability of dormant meristems, adventitious sprouting is not subject to such limitation (support for Hypothesis 1). On the intraspecific level, we found that between intact and injured plants, the relationships to environmental factors often differed, implying that apical meristem damage can indeed change limitations for plant growth (Hypotheses 2 and 3; see also [11]). In our study, however, the effects of environmental factors on plant height, axillary branching and adventitious sprouting were seldom significant. Results consistent with our first and second hypotheses were observed for the effects of competition (measured as plant cover), as, with increasing competition, axillary branching after injury increased whereas adventitious sprouting decreased. Other environmental conditions showed only weak associations with activation of reserve meristems (both axillary and adventitious), and not in the direction predicted by our hypotheses. The number and length of adventitious sprouts were negatively affected by increasing light availability both in intact and injured plants, and spontaneous adventitious sprouting was supported in favorable conditions (high nutrients and moisture), but no effect was found for injured plants (rejecting Hypothesis 3).

### Intraspecific Level

Surprisingly, even some effects that have been frequently reported, i.e. increased branching of plants in high-nutrient or low-competition [29] conditions, were not found in our dataset. One possibility is that different types and intensities of compensatory growth are initiated by different sources and/or timing of damage, and to the extent these damage sources varied among sites, this could have precluded formation of generalized patterns across sites. The absence of such general effects in our study could not have been due to intercorrelations among studied factors, because although high herb layer cover was correlated with soil moisture and nutrients (and not with light!), the relationship was very weak, with less than 2% of variability shared, and significant due only to the large size of our data set (moisture: R^2^ = 0.018, F_1,357_ = 6.55, p = 0.01, nutrients: R^2^ = 0.018, F_1,357_ = 5.433, p = 0.02). On the other hand, adventitious sprouting was supported by high soil nutrients and moisture, but only in intact plants. Interestingly, this outcome was not detected in our previous study [15], in which the pooling of data across injured and intact plants apparently obscured this relationship, with no significant effect of environment found on plants overall.

In the present study, in contrast, high herb layer cover positively affected branching in injured plants and negatively influenced adventitious sprouting. We would expect, based on Aarssen [30], that increasing plant height in competition (found for both injured and intact plants in our study) would be at the expense of branching, but in intact plants, no effect of plant cover on branching was found. However, in agreement with hypotheses 2 and 3, injured plants branched more and showed less adventitious resprouting in high competition. One possible explanation is that high plant cover attracted greater herbivory intensity. Alternatively, community-level disturbance at sites that, at the time of sampling, showed higher plant cover could have occurred earlier than at other sites, with the plants having already recovered from it. Injury to a plant body occurring earlier in a plant’s ontogeny may have a substantially different effect on branching than such injury later in ontogeny [31], [32], [33], with this timing difference perhaps influencing the observed cover values.

Another unexpected finding was the negative effect of high light availability on the number and length of adventitious sprouts for both injured and intact plants. This result indicates that the behaviour of adventitious sprouting differs from the pattern usually observed for axillary branching [29], [30], [34]. The suppression of adventitious sprouts in terms of both number and length by high light conditions was likely due to regulatory signals that prevent uncontrolled sprouting from adventitious buds [35].

In our study, branching and sprouting were not limited by either soil nutrients or competition. This lack of a consistent, general relationship among compensation, competition and nutrients is in accord with the varying results obtained in other studies (i.e., this variation in relationships great enough that neither CCH nor GRM can predict outcomes in more than half of cases, whereas LRM’s predictions are essentially case-specific, see Introduction). Our study did show an important distinction between branching (from axillary buds) and sprouting (from adventitious buds) in the effect of dormant meristem availability on response to injury, in that, with increasing injury severity, the former, but not the latter, was limited by dormant meristem availability. Thus, studies considering only fitness, rather than branching and sprouting are likely to produce an incomplete and potentially misleading picture of plant responses to injury.

Indeed, recognizing the difference in responses between axillary branching and adventitious sprouting can be helpful in further developing our understanding of damage tolerance. It is already known that, in comparison with axillary branching, adventitious sprouting requires more intense stimuli and longer times for shoot development [36]. In the present study, plants damaged only slightly responded by branching from easily activated axillary buds, whereas severely injured plants with only a few axillary buds left resprouted from adventitious buds. This multi-tiered system of damage response can represent a solution to the challenge presented by the fact that although a low threshold for bud activation is favored if damage events occurs only once during a growing season, easy activation renders the new shoots vulnerable to subsequent damage (see [11], [12]). In discussing bud dormancy, Nilsson *et al.* [12] describe bet hedging as a “strategy that does not put all eggs in the same basket … [in which] … meristems are the eggs and shoot types are the baskets”. Using this characterization, plants capable not only of axillary branching, but also of adventitious resprouting, appear to have another proverbial “basket”. They are thus able to employ a more sophisticated bet-hedging strategy than the simple choice of active versus dormant meristems represented in existing models.

Plants with potential bud banks (approximately 10% of plants from Central Europe – [37] have indeed been found widely in ecosystems characterized by recurrent disturbance, such as fire-prone habitats [38], badlands [39], and arable fields [40]. Although herbivory has largely been assumed to be the major driving force behind evolution of compensatory growth responses, it would seem that, in some situations, other sources of AMD could exert considerable selection pressure, and least partially underlie the evolution of adventitious sprouting capability. Moreover, compensatory abilities evolved in response to one source of damage could provide pre-adaptation to other types of damage events. This could be particularly important in shaping preadaptation to anthropogenic disturbance. Thus, for example, plants in arable fields or pastures could be preadapted to cutting based on prior evolutionary history with grazing ungulates, although to the extent that a particular growth response requires a particular signal (e.g. saliva) from an herbivore, that response would not be duplicated.

The differences between phylogenetically corrected (non-significant) and uncorrected (significant) relationships of sprouting and/or branching with some environmental variables are interesting, to the extent that they suggest a phylogenetic influence on these behaviors, given that sprouting ability has been seen as phylogenetically labile [20], [21]. However, the reported evolutionary lability of sprouting has emerged from studies that only considered this ability dichotomously (presence versus absence), suggesting that more in-depth examinations might reveal greater phylogenetic influence.

### Interspecific Level

Surprisingly, for the 22 species, we found almost no differences in branching or sprouting attributable to their position along environmental gradients. The species did occupy a rather narrow extent of the gradients (with lower and upper thirds for Ellenberg indicator values for light at 6.7 and 7.4, respectively; for moisture at 4.4 and 5.3; and for nitrogen at 5.4 and 6.9), but interspecies differences in habitat preferences according to EIVs (tested using ANOVA without phylogenetic correction) were highly significant (*p*<0.0001) and relatively strong (ca. 50% variation in EIVL/EIVM/EIVN explained by species identity). The rather similar environmental niches and shared life-history strategy of our study species likely excluded the possibility of detecting any general trends on the interspecific level.

### Conclusion

Compensatory growth in our study system was limited only by the availability of meristems, and this limitation was overcome by the ability of plants to produce adventitious buds – the potential bud bank – and to sprout from them. We did not find any simple relationship between branching or sprouting and availability of nutrients, soil moisture, or competition across 22 species in 389 populations growing in natural conditions. Thus, the effects of competition and nutrient availability on compensatory responses, in terms of branching and sprouting, to apical meristem damage cannot be generalized.

Future research on evolution of plant tolerance to damage will profit from comparing phylogeny, habitat preferences, and trade-offs of adventitious sprouters with those of other plants. Additionally, it would be worthwhile to experimentally investigate whether particular patterns of compensatory growth (in terms of magnitude and/or type) occur in response to particular AMD sources such as drying or freezing events associated with certain habitats.

## Supporting Information

Appendix S1 **List of the studied species and numbers of sampled populations during 2005 and 2006 field seasons.**
(DOC)
