# Supplementary material for: Potential Bud Bank Responses to Apical Meristem Damage and Environmental Variables: Matching or Complementing Axillary Meristems?
Source: PLoS One. 2014 Feb 6;9(2):e88093. doi: 10.1371/journal.pone.0088093 (PMC3916394; doi:10.1371/journal.pone.0088093)
Supplement: Appendix S1 — List of the studied species and numbers of sampled populations during 2005 and 2006 field seasons. (DOC) [file pone.0088093.s001.doc]

Appendix S1 – List of the studied species and numbers of sampled populations during 2005 and 2006 field seasons.

| Life-History | Species | Family | Populations |
| --- | --- | --- | --- |
| Annual*s* | *Anagallis arvensis* L. | Primulaceae | 30 |
|  | *Arabidopsis thaliana* (L.) Heynh. | Brassicaceae | 30 |
|  | *Euphorbia exigua* L. | Euphorbaceae | 6 |
|  | *Euphorbia helioscopia* L. | Euphorbaceae | 30 |
|  | *Euphorbia peplus* L. | Euphorbaceae | 31 |
|  | *Kickxia spuria* (L.) Dumort. | Scrophulaceae | 3 |
|  | *Kickxia elatine* (L.) Dumort. | Scrophulaceae | 2 |
|  | *Microrrhinum minus* (L.) Fourr. | Scrophulaceae | 31 |
| Biennials | *Arabis glabra* (L.) Bernh. | Brassicaceae | 6 |
|  | *Arabis hirsuta* (L.) Scop. s.str. | Brassicaceae | 2 |
|  | *Alliaria petilotata* (M. Bieb.) Cavara et Grande | Brassicaceae | 27 |
|  | *Barbarea vulgaris* W.T. Aiton | Brassicaceae | 31 |
|  | *Barbarea stricta* Andrz. | Brassicaceae | 10 |
|  | *Daucus carota* L. | Apiacaceae | 35 |
|  | *Diplotaxis muralis* (L.) DC. | Brassicaceae | 1 |
|  | *Isatis tinctoria* L. | Brassicaceae | 2 |
|  | *Jasione montana* L. | Campanaceae | 5 |
|  | *Medicago lupulina* L. | Viciacaceae | 29 |
|  | *Potentilla supina* L. | Rosacaceae | 30 |
|  | *Reseda lutea* L. | Resedacaceae | 15 |
|  | *Reseda luteola* L. | Resedacaceae | 1 |
|  | *Rorippa palustris* (L.) Besser | Brassicaceae | 32 |
